# Radiological assessment of immunotherapy effects and immune checkpoint‐related pneumonitis for lung cancer

**DOI:** 10.1111/jcmm.17895

**Published:** 2023-07-31

**Authors:** Qiongjie Hu, Shaofang Wang, Li Ma, Ziyan Sun, Zilin Liu, Shuang Deng, Jianlin Zhou

**Affiliations:** ^1^ Department of Radiology, Tongji Hospital, Tongji Medical College Huazhong University of Science and Technology Wuhan China; ^2^ Department of Orthopedics, Songzi Hospital Renmin Hospital of Wuhan University Wuhan China; ^3^ Department of Orthopedics Renmin Hospital of Wuhan University Wuhan China

**Keywords:** adverse events, immunotherapy, lung cancer, pneumonia, radiologic, treatment outcome

## Abstract

Immune checkpoint inhibitors (ICIs) therapy have revolutionized advanced lung cancer care. Interestingly, the host responses for patients received ICIs therapy are distinguishing from those with cytotoxic drugs, showing potential initial transient worsening of disease burden, pseudoprogression and delayed time to treatment response. Thus, a new imaging criterion to evaluate the response for immunotherapy should be developed. ICIs treatment is associated with unique adverse events, including potential life‐threatening immune checkpoint inhibitor‐related pneumonitis (ICI‐pneumonitis) if treated patients are not managed promptly. Currently, the diagnosis and clinical management of ICI‐pneumonitis remain challenging. As the clinical manifestation is often nonspecific, computed tomography (CT) scan and X‐ray films play important roles in diagnosis and triage. This article reviews the complications of immunotherapy in lung cancer and illustrates various radiologic patterns of ICI‐pneumonitis. Additionally, it is tried to differentiate ICI‐pneumonitis from other pulmonary pathologies common to lung cancer such as radiation pneumonitis, bacterial pneumonia and coronavirus disease of 2019 (COVID‐19) infection in recent months. Maybe it is challenging to distinguish radiologically but clinical presentation may help.

## 1 BACKGROUND

Global lung cancer incidence has been the second for many years.^1^ However, it becomes the first leading cause of cancer death overall.^1^ Immune checkpoint inhibitors (ICIs) have revolutionized treatment in advanced lung cancer.^2^ Recently studies reported that the benefits of immunotherapy appeared to be safer and better in early lung cancer than those observed in advanced lung cancer, especially with the regard to the regimen of immunotherapy in combination with chemotherapy.^3^, ^4^ However, patients receiving immunotherapy exhibit potential initial transient worsening of disease burden and delayed time to treatment response.^5^ Besides, it is worth noting that series of immune‐related adverse events (irAEs) are distinct from those happen in traditional cancer therapies.^6^ Moreover, the irAEs can involve almost all organ systems, with mainly occurring on single organ system and commonly on the skin, gastrointestinal tract, endocrine system and liver.^7^, ^8^ Immune checkpoint inhibitor‐related pneumonitis (ICI‐pneumonitis) is one of the immune‐related adverse events that sometimes cause lethal outcomes.^9^, ^10^, ^11^ As the clinical manifestation is often nonspecific, computed tomography (CT) scan plays an important role in the diagnosis and triage.

### 1.1 Traditional therapy and immunotherapy for lung cancer

Lung cancer is a public health problem and the leading cause of cancer incidence and mortality worldwide, with an estimated 2.1 million new cases and 1.8 million deaths annually.^12^ The overall 5‐year survival is only 60%–70% due to the poor prognosis.^13^ Surgical operation as one of the traditional treatments, is the recommended treatment for patients with lung cancer at early‐stage. Patients considered inoperable are mainstay treated with chemotherapy and radiotherapy. However, due to tumour‐induced immune failure to recognize tumour antigens and to kill malignant cells, these above traditional therapies deliver suboptimal outcomes.^14^, ^15^ Thus, cancer immunotherapy has been developed to enhance the power of immune system.^16^

Immune checkpoints and ICIs are important concepts in cancer immunotherapy. As main prescribed drugs in current immunotherapies practice, the former immune checkpoints, such as cytotoxic T‐lymphocyte antigen 4 (CTLA‐4) and programmed cell death protein 1 (PD‐1), are usually expressed on the membrane of cytotoxic T cells and could interact with corresponding ligands, critical in maintaining immunologic homeostasis.^2^, ^17^ On the other hand, ICIs could act as monoclonal antibodies and directly block the immune checkpoint‐related cellular pathways on T cells.

Chemotherapy combined with immunotherapy had become to be a new tumour treatment mode recently.^4^, ^18^, ^19^, ^20^ Importantly, these benefits were observed not only when it was used alone with CTLA‐4 inhibitors, PD‐1/PD‐L1 inhibitors, but also in combination with chemotherapy, antivascular drugs, or even in the case of dual immunotherapy agents.^3^, ^20^, ^21^, ^22^ ICIs have changed the treatment landscape of advanced non‐small cell lung cancer (NSCLC). Since Ipilimumab, an anti‐CTLA‐4 antibody, was approved for treating metastatic melanoma patients in 2011, several other clinical types of ICIs related immune drugs eliciting the body's immune system to remove or kill neoplastic cells were also emerged, bringing about effective immunotherapeutic tools for treating multiple cancer types.^2^, ^23^, ^24^ Nivolumab and pembrolizumab targeting at PD‐1 have been subsequently approved for the treatment of metastatic NSCLC.^25^, ^26^ Reports have demonstrated that ICIs, which constantly enrich the therapy methods of cancers, could provide admirable survival benefits for patients with certain tumour diseases compared to traditional agents.^26^, ^27^ Additionally, recent studies had revealed safer benefits of ICIs especially in early lung cancer than those in advanced lung cancer, especially with regard to the regimen of immunotherapy in combination with chemotherapy.^3^, ^4^, ^28^ Novel ICIs agents and combinations are developed at rapid pace and the host responses caused by them in multiple indications still need evaluation.

### 1.2 Imaging response criteria of lung cancer immunotherapy

Immunotherapy is a step forward in cancer care. Early studies have shown great promise for immunotherapy in lung cancer and this modality seems to become part of the future armamentarium for treating this most devastating disease.

Tumour burden, the surrogates of survival or quality of life, is validated and consistent criteria for assessing cancer therapy. Patients receiving immunotherapy can exhibit tumour burden patterns that are not always captured by traditional tumour response criteria. Several key differences in the response patterns of ICIs therapeutic agents compared with traditional tumour treatments include the potential initial transient worsening of disease burden, either through lesion enlargement or the appearance of new lesions (i.e. pseudoprogression), and delayed time to treatment response.^5^, ^29^

As a family member of conventional imaging response criteria, Refined Response Evaluation Criteria in Solid Tumurs version 1.1. (RECIST 1.1) is the most widely used standard for tumour curative effect evaluation.^30^ However, RECIST 1.1 also has shortcomings in the evaluation of treatment response for ICIs therapy, such as leading to the potential for premature cessation of therapy in patients who might otherwise show benefit with therapy. RECIST 1.1 also cannot accurately assess the immunotherapy, such as “delay effect”.^31^ Subsequently, updated treatment response criteria such as the immunerelated response criteria (irRC), has been developed to account for the unique imaging features, also for continuously meeting the needs to standardize and validate response criteria.^31^

**FIGURE 1:**
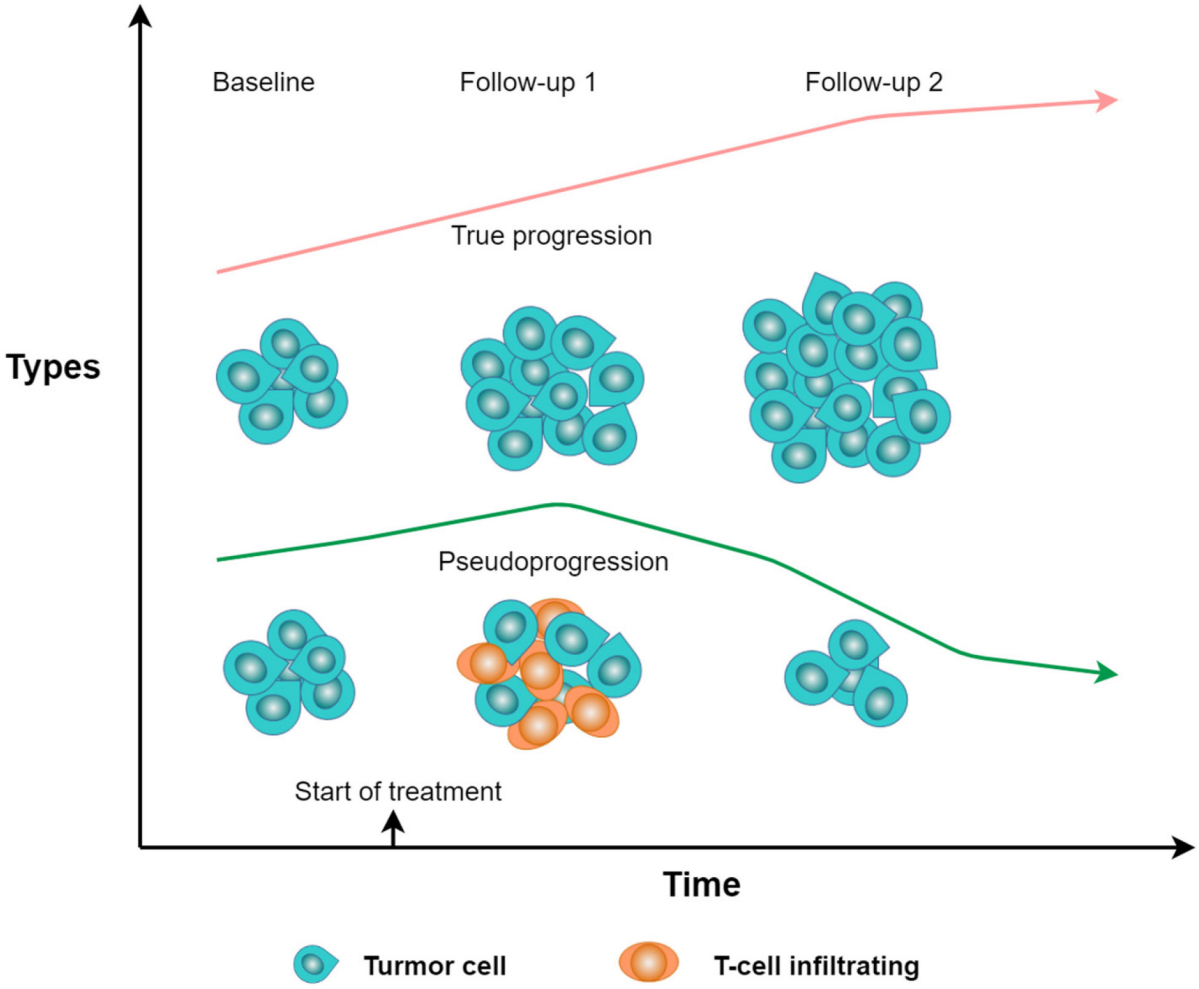
Pictorial illustration depicting tumour progression versus pseudoprogression.

Challenges on differentiating transient pseudoprogression (described as the visualization of new lesions, or an increase in the size of lesions) from true progression prompts the irRC standard production.^32^ IrRC used WHO criteria and modified the alternate definitions of progressive disease (PD) in the overall calculated tumour response to standardize and validate response. In general, irRC recommends follow‐up response assessment every 6–12 weeks, as the same as RECIST 1.1.^5^, ^30^ Response assessments should be done on a calendar schedule and not be affected by delays in therapy. It requires an earlier confirmatory scan, to confirm complete or partial response. Here, we compare the different criteria of immunotherapy’ assessment in detail^30^ as below (Table 1 and Figure 1).

**TABLE 1:** Comparison of response categories between different criteria.^30^

	WTO criteria	RECIST 1.1 criteria	irRC criteria
Measure			
New measurable lesions (≥5 × 5 mm)	PD	PD	Tumour burden
New non‐measurable lesions (≥5 × 5 mm)	PD	PD	Non‐PD
Immune complete response (iCR)	≥ 4 weeks Two consecutive abservation of all the target lesions disappeared	≥ 4 weeks Two consecutive abservation of all the target lesions disappeared	≥ 4 weeks Two consecutive tumour burden disappeared
Immune partial response (iPR)	≥4 weeks Two consecutive observation of sum of the products of diameters (SPD) to reduce 50% or higher from baseline	≥4 weeks Two consecutive observation target lesion diameter combined to reduce 30% or higher	≥4 weeks Two consecutive observation tumour burden by 50% or more
Stable disease (SD)	SPD decrease <50% or increase<25% compare to the baseline, with no new lesions or not clear progress of target lesions	Does not meet the PR tumour or tumour enlargement is not enough to evaluate PD	The tumours burden decrease by 50% or increase by 25%
Progressive disease (PD)	SPD increase ≥25%, and/or the target lesions clear progress, and/or new lesions	Target lesion diameter combined to reduce ≥20%, or appear one or more new lesions	≥ 4 weeks Two consecutive observation of the tumour burden increase ≥25%

## 2 ICI‐PNEUMONITIS

Although therapies of ICIs get ground breaking therapeutic advances and bring lasting clinical benefits,^33^ it is worth noting that series of irAEs are distinct from traditional cancer therapies.^6^ It should also be noted that the toxicity can occur from any time including the outset of ICIs treatment, during treatment, or after treatment discontinued.^34^, ^35^ The irAEs involve almost all organ systems, and often happened on single organ system including the skin, gastrointestinal tract, endocrine system, cardiovascular system and so on.^7^, ^8^, ^36^

ICIs immunotherapy with anti‐CTLA‐4 agents leads to a highest incidence of irAEs (of any grade) of 60%–85%, compared with 39%–70% while treating with anti‐PD‐1/anti‐PD‐L1 agents.^37^, ^38^, ^39^ Fortunately, these irAEs have been reported with less severe for the low grades (Grades 1–2) and higher frequencies.^40^ Dermatological adverse events, usually the first to appear after patients undergone treatment with antibodies that block the anti‐PD‐1/PD‐L1 signalling, are most commonly encountered, whereas other organ toxicities occur less frequently.^41^ There's diversity of performance in these skin toxicities, such as a mild rash or even severe epidermolysis.^38^, ^42^ However, the underlying mechanism is still not fully understood. A hypothesis exists regarding these skin toxicities pointing autoreactive T‐cells, autoantibodies and cytokine toxicities.

Endocrine‐related^43^ adverse events are another common performance of irAEs, affecting thyroid, hypophysis and adrenal glands.^9^, ^33^, ^44^ One of the most frequent irAEs in patients with ICIs treatment is thyroid dysfunction. The incidence of thyroid dysfunction is 7.0% in those treated with anti‐PD‐1 agents,^44^ and become 13.2% while PD‐1 agents are used in combo therapy.^44^, ^45^

ICI‐pneumonitis is one of the immune‐related adverse events that sometimes cause lethal outcomes.^9^, ^10^, ^46^ The onset of ICI‐pneumonitis was variational, occurring weeks to many months after initiation of therapy.^47^, ^48^, ^49^, ^50^ Clinical symptoms may be asymptomatic, or present with non‐specific symptoms of dyspnea, cough, fatigue, hypoxia, chest pain, or hemoptysis.^51^ Hypoxia may progress rapidly, leading to respiratory failure, even life‐threatening.

### 2.1 Risk factors

The frequency of ICI‐pneumonitis is mainly dependent on the used agents, administered dose and exposure time as well as the intrinsic risk factors of patients.^52^ In NSCLC patients, the incidence of ICI‐pneumonitis was reported as high as 7%–19% in some clinical settings in recent reports.^53^, ^54^ Other studies reported more treatment‐related deaths of pneumonitis in NSCLC patients, but similar rates of Grades 3–4 pneumonitis compared with other tumour types. Overall incidence of pneumonitis associated with anti‐PD‐1/PDL1 agents was approximately 3% in a meta‐analyses published by Wang et al. in 2017,^55^ and the incidence of high‐grade pneumonitis was 1.53%.^56^ However, it's exceptionally rare in patients receiving anti‐CTLA4 antibodies. Moreover, onset tended to occur earlier and more frequent (5%–10%) in patients who received combination therapy than in those who received monotherapy.^51^, ^57^ Combination therapy with ipilimumab plus nivolumab resulted in the highest incidence of pneumonitis (5%–10%), and 2% were reported to be Grade 3 or 4 events.^57^ After the high radiation dose of radiotherapy (RT), incidence of ICI‐pneumonitis increased.^58^ Besides, the history of chronic lung disease, such as interstitial lung disease, especially lung fibrosis, chronic obstructive pulmonary disease (COPD) and asthma, was independently associated with an increased incidence of ICI‐pneumonitis.^51^, ^59^, ^60^ Thomas HMT et al. reported the role of novel functional lung radiomics for pneumonitis risk stratification in locally advanced NSCLC and found that the strongest predictor of ICI‐pneumonitis was the presence of baseline COPD.^61^ And some reported that greater smoking history, lower pre‐treatment lung function, are risk factors associated with ICI‐pneumonitis.^62^

### 2.2 Diagnosis and management

According to the American Society of Clinical Oncology Clinical Practice Guideline, the diagnostic work‐up should include Chest CT, X‐ray films and pulse oximetry (hypoxia, pulse oximetry <90%).^63^ On chest imaging, typical findings of pneumonitis include ground‐glass opacities (GGO) or/and consolidations, predominantly to be focal in the lower lobes, which is different from targeted agents related diffuse pneumonitis.^63^, ^64^ Recent reports reviewed several non‐typical findings, such as subpleural micronodular opacities, hilar lymphadenopathy and pleural effusions. Moreover, infection through nasal, sputum, blood and urine should be excluded.

To standardize management ICI‐related pneumonitis, the ICI‐pneumonitis are recommended to grade severity according to National Comprehensive Cancer Network (NCCN) Clinical Practice Guidelines in Oncology and the National Cancer Institute Common Terminology Criteria for Adverse Events (CTCAE). Early recognition of toxicity and institution of management algorithms of ICI‐pneumonitis are key to ensure patient safety. Here, we review the common toxicities and provide recommendations on their management on different grades of ICI‐pneumonitis which are usually classified by the combination of imaging findings and/or clinical symptoms as described below (Table 2).^26^, ^65^, ^66^ Fortunately, most of the ICI‐pneumonitis are manageable, and in some cases, these toxicities are fulminant, fatal and lead to the withdrawal of treatment depending on the different classification. Most cases of ICI‐ pneumonitis resolve or improve with corticosteroids; however, a small proportion of patients may develop recurrent pneumonitis when rechallenged with ICIs.

**TABLE 2:** Management of ICI‐pneumonitis.^26^, ^65^

Grade	Grade definition	Management	Disposition and follow‐up
1	Asymptomatic or mildly symptomatic	Continue immunotherapy with close monitoring, with the exception of some neurologic, hematologic, and cardiac toxicities. Monitor symptoms and oxygen saturation	Update and establish follow‐up with outpatient oncologist
2	Moderately symptomatic or have symptoms that limit age‐appropriate instrumental activities of daily living	Holding immunotherapy Low dose of 1 mg/kg/day of prednisone or equivalent Consider prophylactic antibiotics for opportunistic infections Consider resuming when symptoms and/or laboratory values revert to Grade 1.	Referral to corresponding department Educate patient to return for worsening symptoms
3	Severe symptoms or medically significant, but not immediately life threatening	Permanently discontinue immunotherapy High‐dose of 2 mg/kg/day methylprednisolone Consider prophylactic antibiotics for opportunistic infections	Admit to hospital for further evaluation and work‐up
4	Life‐threatening necessitating urgent intervention.	Permanently discontinue immunotherapy Consider for infliximab, cyclophosphamide, IVIG, or mycophenolate High‐dose of 2 mg/kg/day prednisone or methylprednisolone	Admit to ICU for further evaluation and work‐up

#### 2.2.1 Radiographic and ICI‐pneumonitis

Chest CT better depicts subtle changes and differentiates pneumonitis. The role of another common imaging method, PET in the diagnosis and follow‐up of ICI‐pneumonitis is unclear, because PET lacks in diagnostic specificity in hypermetabolic activity with ICI‐pneumonitis, malignancy and infectious processes.^67^ GGO or consolidations in the peribronchovascular distribution, predominantly in the lower lobes, are common manifestations on chest CT imaging.^59^ Therefore, new or progressive pulmonary GGO or consolidation in patients receiving immunotherapy, especially when combined with hypoxia (pulse oximetry <90%), should be suggestive of ICI‐pneumonitis.^68^ Interlobular septal thickening, intralobular lines, and crazy paving were also seen. The lesions are symmetrical or asymmetrically distributed.

In addition to typical findings of pneumonitis, subpleural micronodular opacities and hilar lymphadenopathy, which seem like sarcoid granulomatous reactions, as well as pleural effusions have been reported in ICI‐pneumonitis.^69^, ^70^, ^71^, ^72^ As they may mimic disease progression on imaging and examination, it is prudent to be aware of the possibility of such immune‐related pulmonary reactions. Biopsy may assist in confirming the diagnosis. Ipilimumab induced sarcoid‐like granulomatous reaction with macrophages surrounded by inflammation.^73^, ^74^

For ICI‐pneumonitis recrudescence, consolidative or GGO in the peribronchovascular distribution was similar to the initial episode in most cases on chest CT, and re‐emergence involved new airspace opacities in previously uninvolved was also seen.^68^

#### 2.2.2 CT patterns of ICI‐pneumonitis

A large retrospective study described a spectrum of radiologic manifestations of ICI‐pneumonitis, which systematically assessed the presence, distribution and extent of each feature on chest CT scans. There are several suggestive radiographic patterns categorized according to the classifications of interstitial pneumonias in the American Thoracic Society/European Respiratory Society: (i) Organizing pneumonia (OP) pattern, (ii) non‐specific interstitial pneumonia (NSIP) pattern, (iii) hypersensitivity pneumonitis (HP) pattern, (iv) acute interstitial pneumonia (AIP) pattern, (v) bronchiolitis.

**FIGURE 2:**
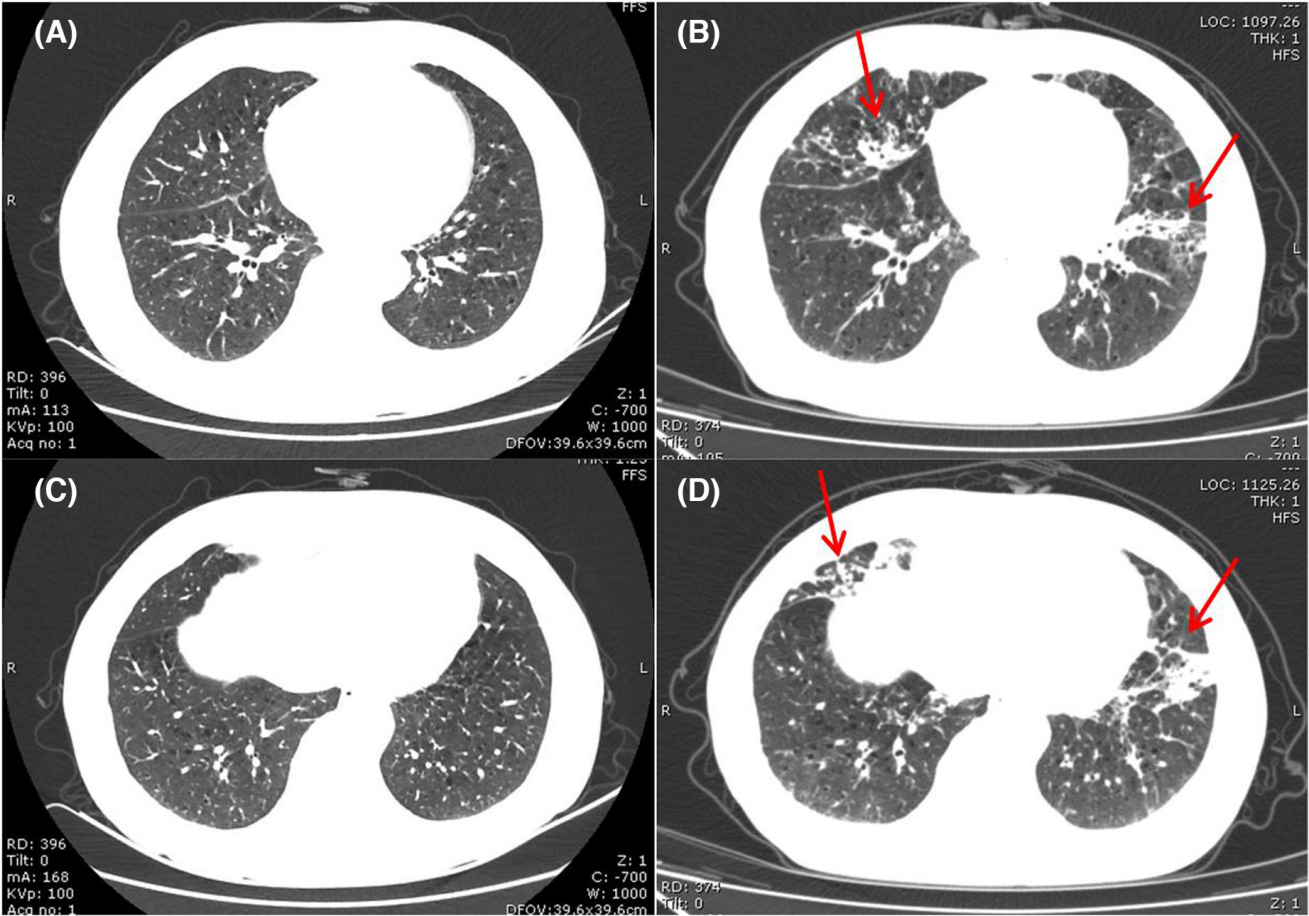
OP pattern in a 69‐year‐old man who underwent Keytruda therapy for stage IV lung Squamous cell carcinoma. (A) and (C) A baseline chest CT image before starting immunotherapy. (B) and (D) Axial chest CT image obtained 3 months after starting immune checkpoint inhibitor therapy shows multifocal subpleural mid‐ and lower‐lung consolidations (arrows).

OP pattern^75^ is the most frequent observation of ICI‐pneumonitis, which manifests as consolidative or GGO or a combination of both, multifocal peribronchial and subpleural, often with a lower‐lung predominance. Air bronchograms with or without bronchial dilatation were frequently observed. Reversed halo or atoll sign has also been reported in ICIs therapy‐related pneumonitis.^76^ The lesions changed location or configuration over time were also reported (Figure 2).

**FIGURE 3:**
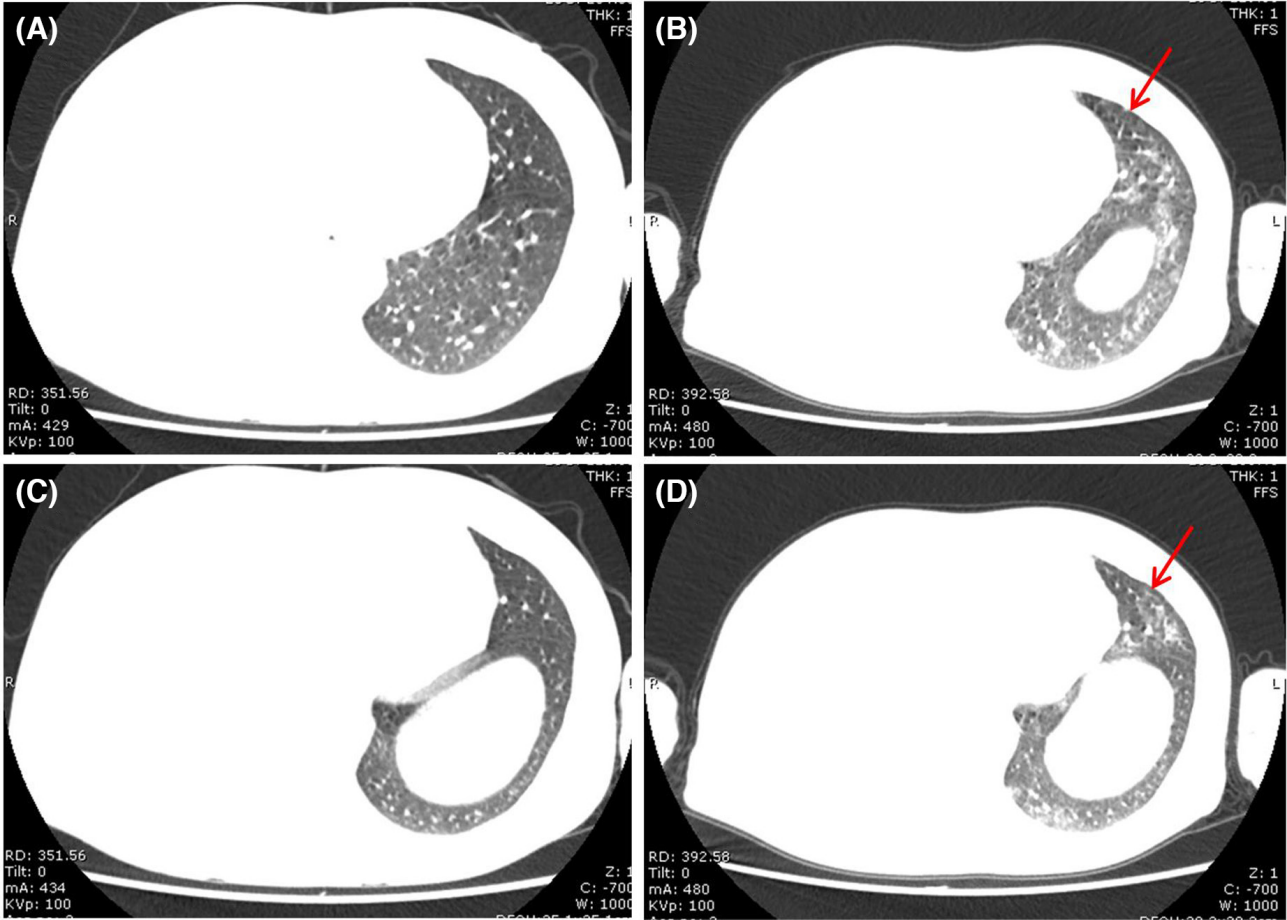
NSIP pattern in a 54‐year‐old man who underwent keytruda therapy for stage IV large‐cell lung cancer. (A and C) Baseline chest CT images before immunotherapy was initiated. (B and D) Axial chest CT image obtained 3 months after starting immunotherapy shows bilateral lower lobe ground‐glass and reticular opacities (arrows).

NSIP pattern, which is the second most commonly described pattern of ICI‐pneumonitis, predominantly manifests with subpleural GGO and interstitial thickening with lower lobe predominance. However, changes of fibrotic NSIP including lower lobe volume loss and traction bronchiectasis have not been reported in ICI‐pneumonitis. It is associated with lower grade symptoms (Figure 3).

**FIGURE 4:**
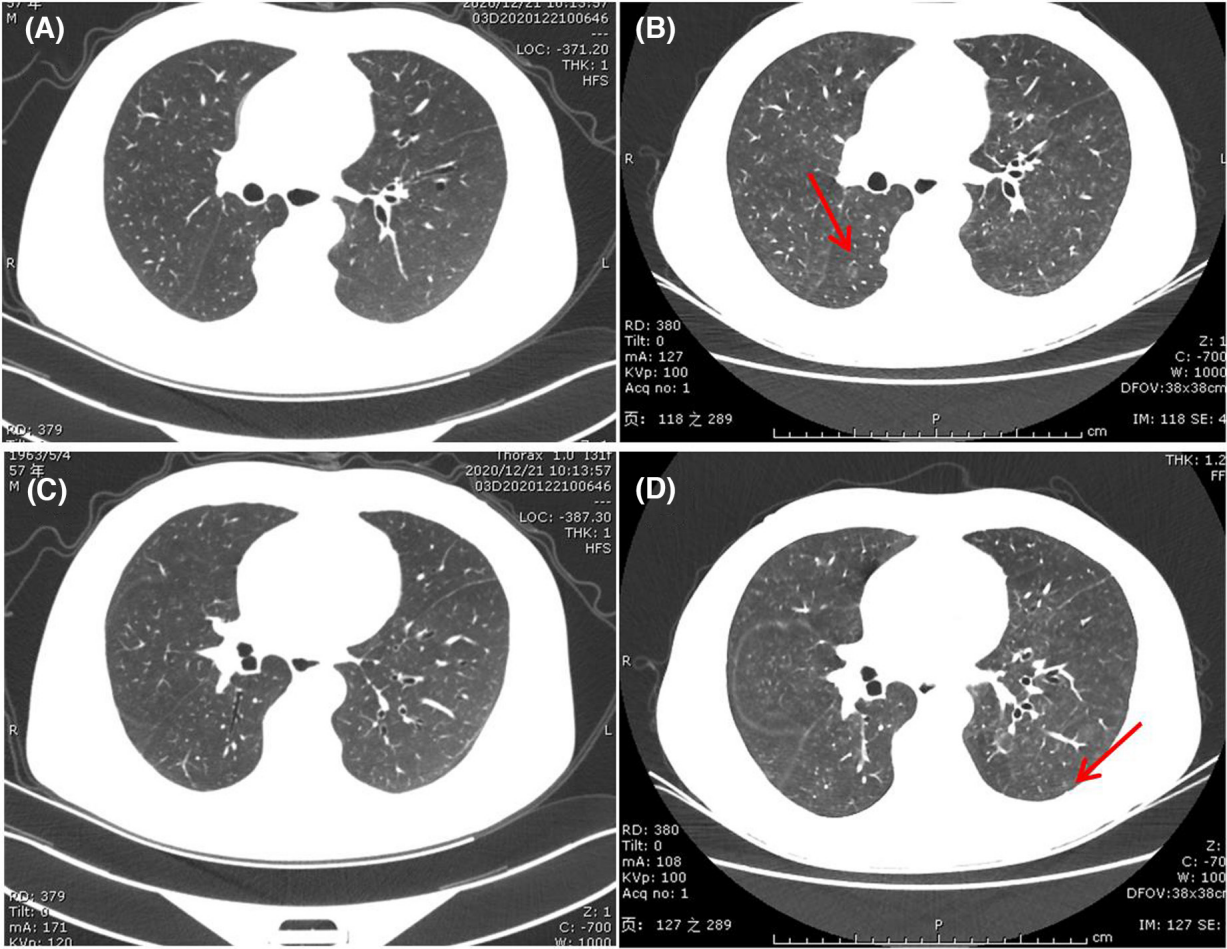
HP pattern in a 58‐year‐old man who underwent nivolumab therapy for stage IV lung adenocarcinoma. (A and C) A baseline chest CT image before starting immunotherapy (not shown) showed no airspace abnormalities. (B and D) Axial chest CT images obtained 5 months after starting nivolumab therapy shows diffuse centrilobular ground‐glass nodules (arrows).

HP pattern appears as characterized by predominantly centrilobular ground glass nodular opacities, with or without accompanied by air trapping. These findings mirror subacute HP in other settings. HP pattern is also associated with lower grade symptoms (Grade 1) (Figure 4).

**FIGURE 5:**
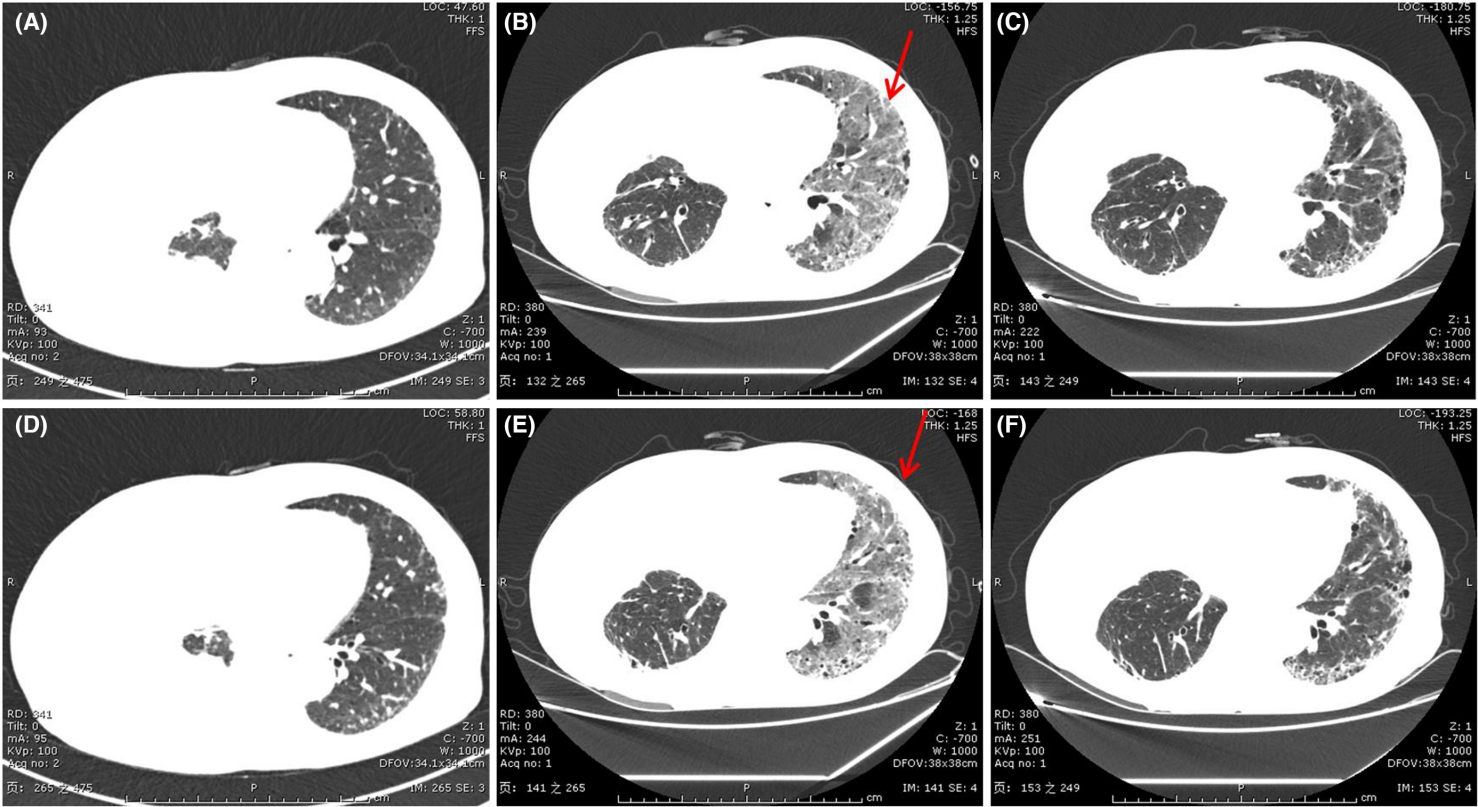
AIP pattern in a 70‐year‐old man undergoing keytruda therapy for stage IV lung adenocarcinoma. (A and D) Baseline axial chest CT images obtained before starting immunotherapy shows multiple lung nodules and masses in the right lung. (B and E) 2 months after starting keytruda therapy, the patient presented with severely worsening dyspnea without fever. Axial chest CT image obtained 2 days later shows diffuse ground glass opacities throughout left lung. (C and F) Axial chest CT image obtained half of month later after administering steroid therapy shows remarkable absorption on image.

AIP pattern is not a prevalent pattern of ICI‐pneumonitis, although it is most frequently associated with high grade pneumonitis and result in significant morbidity and mortality. Findings of AIP pattern included by diffuse consolidative and GGO throughout both lungs, sometimes the entirety of the lungs, which may be associated with bronchial dilatation. The ‘crazy‐paving’ pattern may also be depicted. (Figure 5).

**FIGURE. 6:**
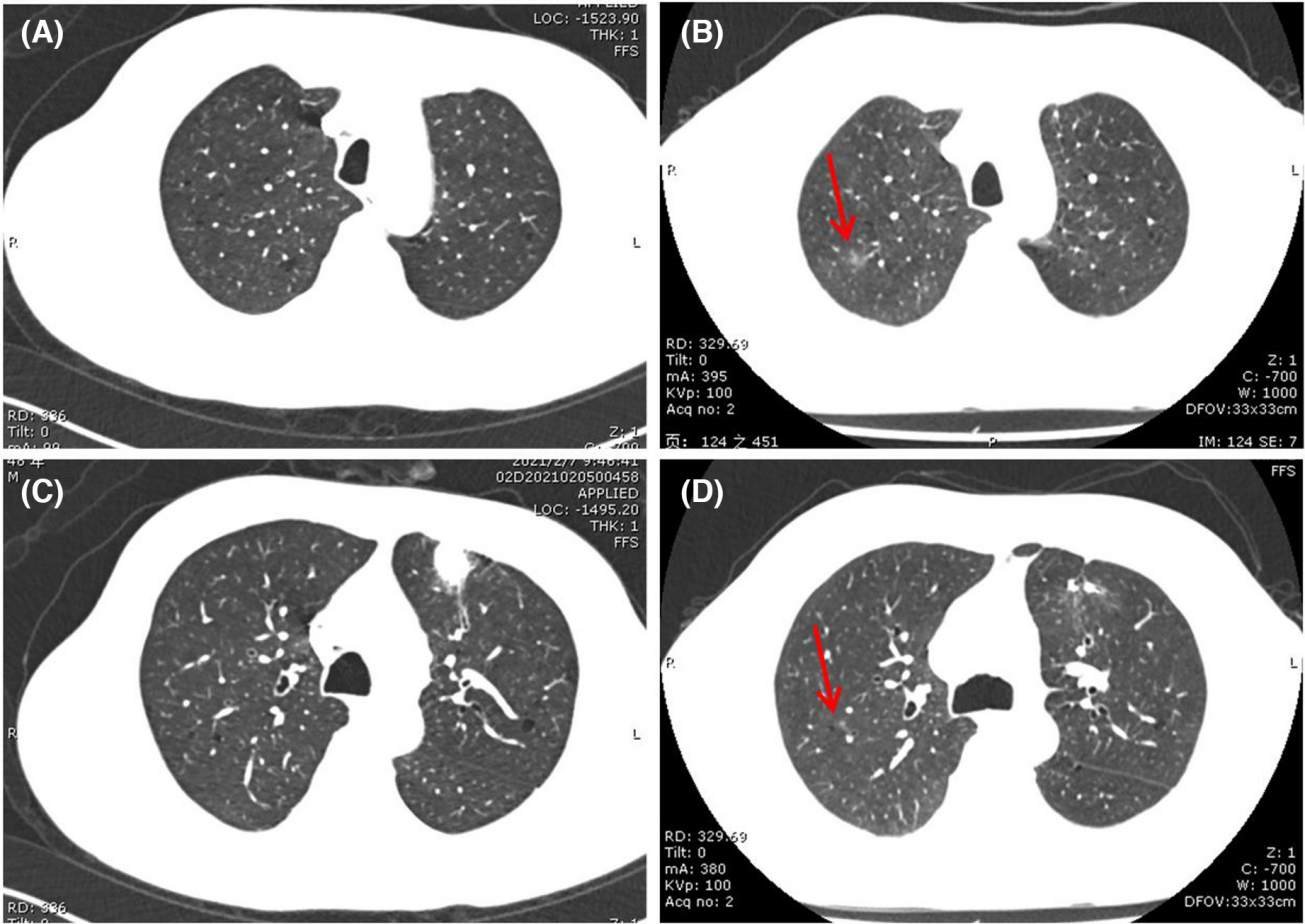
Bronchiolitis pattern in a 48‐year‐old man who underwent keytruda therapy for stage IV lung adenocarcinoma. (A and C) The baseline chest CT images before starting immunotherapy was initiated. (B and D) Axial chest CT images obtained 4 months after starting keytruda therapy shows centrilobular nodularity.

Bronchiolitis pattern is characterized by centrilobular nodularity with or without a tree‐in‐bud pattern. Adjacent bronchial wall thickening is also seen. (Figure 6).

### 2.3 Differential diagnosis

The differential diagnosis between ICI‐pneumonitis and other types of pneumonitis remains challenging. Radiologists should try to differentiate ICI‐pneumonitis from other pulmonary pathologies common to oncology patients such as acute viral infections (specially coronavirus disease of 2019 (COVID‐19) infection), radiation pneumonitis, early pulmonary edema, immune‐related tumour inflammation, or tumour progression and so on.^50^, ^77^, ^78^, ^79^ Recent finding suggested that using the qualitative radiologic features (e.g. number of involved lobes, ground‐glass opacity, consolidation, etc.) may help in the diagnosis.^80^

#### 2.3.1 Differential diagnosis: ICI‐pneumonitis and acute viral infections.

Many advanced lung cancer patients continue to receive regular immunotherapy, such as CTLA‐4, PD‐1, and PDL‐1 ICIs, as salvage therapies even during the acute viral infections such as current COVID‐19 pandemic.^81^, ^82^ Radiographic presentations including GGO, consolidations and reticular opacities in the peribronchovascular distribution were seen on chest CT of ICI‐pneumonitis.^68^ In contrast, the typical CT findings of COVID‐19 pneumonia are not in the peribronchovascular distribution. Bilateral multiple lobular consolidations and GGO, predominantly in the lower lobes, are common manifestations of both ICI‐pneumonitis and COVID‐19 pneumonia on chest CT imaging. Therefore, ICI‐pneumonitis are hard to distinguish from COVID‐19 infection due to their similar radiographic and clinical presentations but different therapy strategies sometimes. It is necessary to combine the results of sputum and serum aetiology, even the more reliable etiological detection of deep sputum specimens.

#### 2.3.2 Differential diagnosis: ICI‐pneumonitis and radiation pneumonitis

Nowadays, immunotherapy after chemoradiotherapy has become the standard therapeutic strategy for unresectable advanced NSCLC and may induce adverse events, including both ICI‐pneumonitis and radiation pneumonitis (RP). And differentiating between ICI‐pneumonitis and RP has significant implications for clinical management.^83^, ^84^ Radiation pneumonitis (RP) usually occurs at 2–6 months after chest radiotherapy. In contrast, the onset median time of ICI‐pneumonitis is 2.8 months (9 days to 19.2 months).^85^ Most RP usually involved area of irradiated lung, involved fewer lobes of the lung and had sharp border with or without respiratory symptoms. In contrast, bilateral multiple lobular GGO, consolidations and reticular opacities in the peribronchovascular distribution, predominantly in the lower lobes, are common manifestations of ICI‐pneumonitis on chest CT imaging. Compared with RP, ICI‐pneumonitis tends to be bilateral, involves more lobes of the lung, and less has sharp borders.^62^ Occasionally, injury is found outside the field of radiotherapy, and can be diagnosed as radiotherapy‐related organized pneumonitis.^68^ For patients with a history of lung radiotherapy, RP should be of concern when new lesions occur during ICIs treatment. In addtion, several studies reported that the radiomic analysis of CT images for differentiating between ICI‐pneumonitis and RP in lung cancer.^62^, ^86^, ^87^

#### 2.3.3 Tumour progression or pseudoprogression?

Patients with true tumour progression always show clinical worsening of symptoms. Immune related criteria confirmed a true progression (at least a new measurable lesions include a size of ≥5 × 5 mm) by a consecutive imaging (CT/MR) assessment at least 4 weeks from the onset time of first administration.^88^ However, T cell infiltration into tumour deposition induced new lesions can be undetectable radiologically at baseline scans. Such an unconventional imaging pattern of tumour response that lacks response to immunosuppressive therapy should not be categorized as progression and the appropriate designation of those types of response is pseudoprogression.^88^ Patients manifested with pseudoprogression are usually asymptomatic, while tumours initially exhibit features of transitory enlargement of lesions, followed by a subsequent radiologic tumour response which is evident on serial imaging with sustained therapy.^89^, ^90^, ^91^ Previous studies have established an approximately 6% incidence of pseudoprogression, with different types of tumour and immunotherapy.^91^, ^92^ A short‐term follow‐up in 4–6 weeks could generally confirm whether it's pseudoprogression or true in these patients.^93^

#### 2.3.4 Differential diagnosis: ICI‐pneumonitis and others

New consolidative or GGO or the reverse halo sign can also be seen with fungal pneumonias,^94^ such as invasive aspergillosis. Additionally, HP pattern of ICI‐pneumonitis is indistinguishable from Allergic alveolitis (allergen exposure, classically birds), and detailed exposure and occupational histories should be sought. Likewise, cardiogenic and non‐cardiogenic pulmonary edema can mimic the AIP/acute respiratory distress syndrome (ARDS) pattern.^95^

However, no pattern was considered pathognomonic, and all radiologic subtypes are diseases that mimic the radiographic patterns of ICI‐pneumonitis.^47^, ^67^, ^96^, ^97^ Imaging findings are non‐specific and can overlap with infectious pneumonia or worsening metastatic disease. Recent report discussed that radiomic biomarkers of computed tomography imaging may support the differential diagnosis of ICI‐pneumonitis and others in patients with NSCLC receiving immunotherapy.^80^ The diagnosis of ICI‐pneumonitis takes the way of exclusion, and a bronchoscopy or bronchoalveolar lavage (BAL) is recommended in cases. A BAL with high lymphocyte infiltration and the inverted CD4/CD8 ratio were expected. A BAL with an inconclusive result, biopsy of histopathology findings of trans‐bronchial include diffuse lymphocytic infiltrates.^74^ However, there are no specific histologic findings for ICI‐pneumonitis currently.^67^ Maybe it is challenging to distinguish radiologically but clinical presentation may help.

## 3 CONCLUSION

With the significant development of novel systemic therapies, the success of ICIs treatment is indisputable in the treatment of lung cancer and other malignancies. Significantly improved outcomes have been demonstrated in certain tumours specially with better tolerance than chemotherapy. However, with the increasing use of ICIs and more cases of adverse events, awareness of the imaging and clinical manifestations of irAEs and response assessment in both trial and clinical practice settings is important in avoiding misinterpretation as progression of disease and informing management decisions with respect to continuation of immunotherapy. In this review, we discussed update imaging response criteria developed and associated with unique adverse events, special for potentially life‐threatening ICI‐pneumonitis. Additionally, as the view of radiologists, we showed differences between ICI‐pneumonitis and other common pulmonary diseases, which might be helpful for clinicians to make appropriate management of oncology patients, while the better management still require further studies.

## AUTHOR CONTRIBUTIONS

**Qiongjie Hu:** Conceptualization (supporting); data curation (lead); investigation (lead); methodology (supporting); project administration (supporting); resources (supporting); visualization (supporting); writing – original draft (supporting); writing – review and editing (supporting). **Shaofang Wang:** Conceptualization (supporting); data curation (lead); formal analysis (lead); investigation (supporting); methodology (supporting); supervision (supporting); visualization (supporting); writing – original draft (lead); writing – review and editing (supporting). **Li Ma:** Investigation (supporting); methodology (supporting); project administration (supporting); visualization (supporting); writing – original draft (supporting). **Ziyan Sun:** Investigation (supporting); methodology (supporting); validation (supporting); writing – original draft (supporting); writing – review and editing (supporting). **Zilin Liu:** Data curation (supporting); software (supporting); writing – original draft (supporting). **Shuang Deng:** Formal analysis (supporting); methodology (supporting); software (supporting); visualization (supporting). **Jianlin Zhou:** Conceptualization (lead); formal analysis (supporting); investigation (supporting); methodology (supporting); project administration (lead); resources (supporting); supervision (lead); writing – review and editing (supporting).

## CONFLICT OF INTEREST STATEMENT

The authors of this article declare no relationships with any companies whose products or services may be related to the subject matter of the article.

## CONSENT FOR PUBLICATION

All authors have reviewed the final version of the manuscript and approved it for publication.

## Data Availability

The data that support the findings of this study are available from the corresponding author upon reasonable request.
